# The baroreceptor reflex brought to life outside the classroom – an e-learning based asynchronous laboratory class using a non-supervised modified Active Standing Test

**DOI:** 10.1186/s12909-022-03573-7

**Published:** 2022-07-01

**Authors:** Tobias Heinrich, Susanne Sehner, Isabel Wageringel, Heimo Ehmke, Alexander Peter Schwoerer

**Affiliations:** 1 Department of Cellular and Integrative Physiology, University Medical Centre Hamburg-Eppendorf, Martinistraße 52, 20246 Hamburg, Germany; 2 Department of Medical Biometry and Epidemiology, University Medical Centre Hamburg-Eppendorf, Martinistraße 52, 20246 Hamburg, Germany; 3 DZHK (German Centre for Cardiovascular Research), partner site Hamburg/Kiel/Lübeck, Hamburg, Germany

**Keywords:** Physiology, Teaching, Education, Orthostasis, Active Standing Test, Schellong test, e-learning, Laboratory class, Digital transition, Baroreceptor reflex

## Abstract

**Background:**

E-learning based laboratory classes can replace or enhance in-classroom laboratories. They typically offer temporal flexibility, self-determined learning speed, repeatability and do not require supervision or face-to-face contact. The aim of this feasibility study was to investigate whether the established in-classroom laboratory class on the baroreceptor reflex (BRR) can be transformed into a new e-learning based asynchronous laboratory class for untrained, non-supervised students without medical equipment. The BRR is a fundamental cardiovascular process which is regularly visualized in physiology during in-classroom laboratories by a student-performed Active Standing Test (AST). During this voluntary provocation of orthostatic stress, the BRR reliably causes a solid rise in heart rate (HR) and a stabilization or even increase in blood pressure (BP).

**Methods:**

The conventional AST was modified by omission of BP measurements which would require medical devices and was embedded into a framework of interactive digital material allowing independent student performance. With specific adaptions, this instrument was implemented to 1st and 2nd year curricula of human medicine, dental medicine, midwifery and pharmacy. An audience response system was used to collect the students’ data on HR, epidemiology, technical problems, satisfaction and orthostatic symptoms. As primary outcome, we investigated the students’ correct performance of the modified AST regarding textbook conformity of the HR data. Secondary outcomes included technical feasibility, the students’ satisfaction and consistency of HR data within predefined subgroups (e.g., gender, curricula). Descriptive statistics are reported.

**Results:**

The class was completed by 217 students (mean age: 23 ± 8 [SD], 81% female, 19% male). Mean reported rise of HR during standing was ~ 20 bpm (~ 30%) which is highly concordant to textbooks. Reported feasibility (~ 80% negated any technical issues) and students’ satisfaction (4.4 on 5-point Likert-scale) were high. The HR data were consistent within the subgroups.

**Conclusion:**

This study demonstrates that the highly relevant BRR can be successfully addressed in an e-learning based asynchronous laboratory class implementing a non-supervised AST restricted to HR measurements embedded in digital material. The robust HR response and the adjustable complexity allow an application to different healthcare-related curricula. This class, therefore, provides a broad audience access to a fundamental concept of cardiovascular physiology.

**Supplementary Information:**

The online version contains supplementary material available at 10.1186/s12909-022-03573-7.

## Background

In-classroom laboratory classes form a fundamental part of teaching physiology in all healthcare-related curricula. They improve procedural, scientific and clinical skills [1, 2], fortify knowledge by application [3] and increase student’s motivation [4]. Students frequently think that “laboratories bring the theory to life and make it real” and that lab classes help learning physiology [5]. These benefits rely on student-performed experiments, data acquisition and analysis [6, 7]. Laboratory classes, therefore, require infrastructure, technical resources, personal efforts and curricular time slots; all factors may be deficient in several settings [8–11].

The digital transition in healthcare-related curricula experienced an additional boost in the COVID-19 pandemic [1, 12–25]. Recent publications on digitalized practical courses use highly heterogenous concepts regarding methodology, didactics and synchronicity of execution [15–25]. Asynchronous e-learning formats are characterized by independent provision and receipt of didactic material. They combine easy distribution of learning materials with temporal and spatial flexibility, enable adaptation to individual learning paces and allow repetitions whenever needed [14]. However, non-supervised experiments require a very clear, unambiguous and motivating framework guiding the students through the theoretical contents, the experimental procedure, the interpretation of the results as well as through the physiological and clinical implications. As uncommented incorrect data can reduce motivation and provoke misconceptions, it is particularly relevant in a non-supervised setting, that student-generated data are concordant to textbooks.

The baroreceptor reflex (BRR) is a fundamental mechanism of cardiovascular physiology which ensures short-term regulation of arterial blood pressure (BP). In the standing position (orthostasis), baroreceptors detect a drop in blood pressure and increase the sympathetic and decrease the parasympathetic tonus. A compensating rise of total peripheral resistance and heart rate (HR) stabilizes or increases arterial pressure [26]. A variety of tests are established to assess this BRR-mediated cardiovascular response in healthy subjects or patients [27–29]. The Active Standing Test (AST) combines feasibility with a high diagnostic sensitivity, validity and reliability [29–35]. It consists of a voluntary provocation of orthostatic stress by a change from supine position to standing, facultatively followed by a second recumbent phase. The cardiovascular response can be assessed by intermittent or continuous recordings of HR and BP with techniques of differing complexity [30, 31]. In physiology laboratory classes, a supervised, student-performed AST is a reliable instrument to visualize the BRR-mediated response to orthostasis. The HR and BP data in combination with frequently experienced orthostatic symptoms foster understanding of the underlying physiology following the concept of experiential learning [30, 32].

Currently, it is unclear, whether the AST can be implemented into an asynchronous e-learning based laboratory class. The absence of a personal introduction and supervision may impair the correct performance of the AST and consequently the data quality and didactical value of the experiment. Moreover, a performance of the AST by a single person instead of a small group and the deficiency of blood pressure cuffs in most households could compromise the transferability of the AST to remote teaching.

Aim of this feasibility study was to investigate whether the established in-classroom laboratory class on the BRR can be transformed into a new e-learning based asynchronous laboratory class for untrained non-supervised students without medical equipment and to report the data. We assumed that the assessment of BP may not be essential for a didactical illustration of the BRR. Therefore, we modified the conventional AST (cAST) by omission of BP measurements and focused on the HR response. This facilitated a performance by individual students. The modified AST (mAST) was embedded into structure providing interactive material to enable a non-supervised performance. This new instrument was applied to 1st and 2nd year curricula of human medicine, dental medicine, midwifery and pharmacy. An audience response system was used to collect the students’ data on HR, epidemiology, technical problems, satisfaction and orthostatic symptoms. As primary outcome, we investigated the students’ correct performance of the mAST with respect to textbook conformity of the HR data. Secondary outcomes were the frequency of orthostatic symptoms, the reported technical feasibility, the consistency of the results within the predefined subgroups separated for gender and the four involved healthcare-related curricula and the students’ satisfaction. Descriptive statistics are reported for all outcomes.

## Methods

### Participants

The newly designed laboratory class was embedded into the cardiovascular physiology modules of human medicine (1^st^ year), dental medicine (1^st^ year), midwifery (1^st^ year) and pharmacy (2^nd^ year) during the COVID-19 pandemic between June 2020 and February 2021 at the medical school Hamburg-Eppendorf, Germany.

### Instrument

Our instrument for the acquisition of HR data during orthostasis was a modified version of the cAST. The cAST is a highly established diagnostical test to assess the HR and BP response to orthostasis which combines a high reliability with a high sensitivity and validity for the detection of orthostatic dysregulations [29–31, 33–36]. The cAST was modified for its didactical use and for the specific requirements of a non-supervised performance by individual students at home without blood pressure cuffs. The positional maneuver remained unchanged, whereas the BP measurements were omitted. HR was intermittently assessed by manual radial palpation. Due to its simplicity, this is a highly established and reliable technique for HR measurements which can also be employed by non-professionals following a brief introduction [37–39]. The mAST was combined with a detailed written step-by-step instruction. This instrument was implemented to our class to provoke and measure the BRR-mediated rise of HR during orthostasis.

### Procedure

**Fig. 1:**
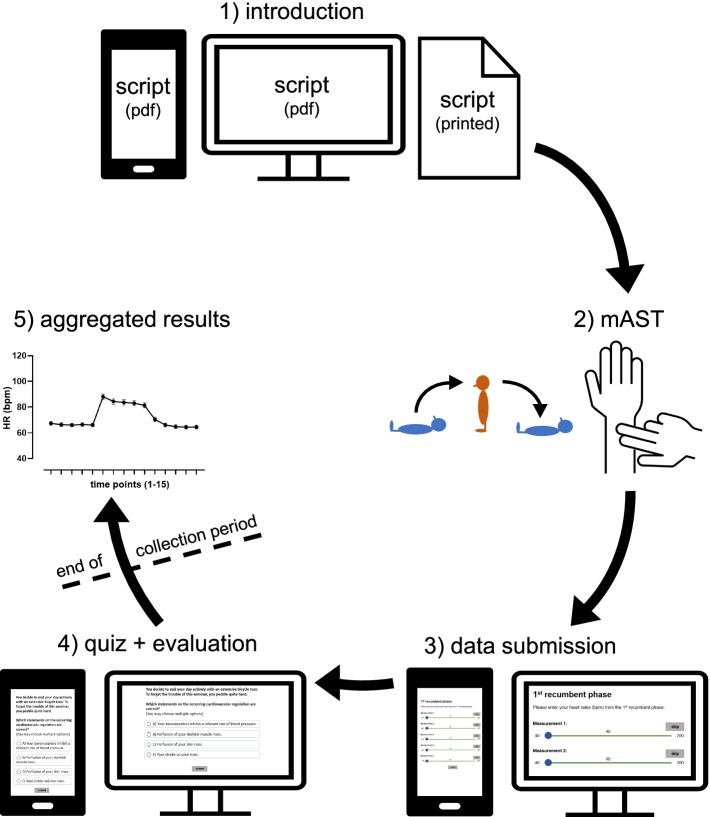
Course structure. The laboratory class consisted of five successive steps for the students. Step 1) introduction: download and reading of the introduction and instructions (digital script), step 2) mAST: performance of the experiment with its three phases (1^st^ recumbent phase, standing phase, 2^nd^ recumbent phase), step 3) data submission: upload of the experimental data with an audience response system, step 4) quiz + evaluation: performance of the post-quiz and evaluation, step 5) aggregated results (after collection period): comparison of the individual measurements with the aggregated data of the cohort

Participation in the laboratory class and submission of the experimental data was highly encouraged but not compulsory and did not have any impact on the students’ grades. Prior to the laboratory class, all students attended the regular physiology lectures and seminars of their specific curriculum. The remote laboratory class could be freely attended online within a specific period (e.g., three weeks following the initial lecture). The laboratory class followed a 5-step approach (Fig. 1). For participation, students needed an end device with an active internet connection and, if necessary, pen and paper for notetaking. Depending on the individual pace and the extent of the provided materials for the specific module, students had to schedule approximately 60 min for the entire laboratory class.

- Step 1), introduction. A digital script was distributed via the e-learning platform. To reduce barriers, the text in the script was easily comprehensible and followed a step-by-step chronology. It included details on the clinical background and on the underlying physiology, stepwise instructions (Additional file 1) on the experimental procedure, a results table (digitally editable or printable) and a link to the audience response system for data submission and the post-quiz.
- Step 2), mAST. The mAST could be performed by the students on themselves or on another person. Participants were advised not to talk during the procedure and to perform in a quiet environment. After an initial resting phase of 5 min, HR was measured every minute during the three phases á 5 min: 1) 1^st^ recumbent phase 2) standing phase 3) 2^nd^ recumbent phase. For standing up, subjects were explicitly advised to avoid unnecessary movements and, once standing, to reduce any further activity to reduce the effects of the muscle pump. The HRs during the 15 time points were documented in the results table of the script.
- Step 3), data submission. Following offline documentation, students submitted their data sets using an audience response system. A direct link and QR-code were embedded in the script. In addition to the experimental results, students were asked about technical issues, career, age, gender and orthostatic symptoms in the past and during the experiment. Due to technical issues and a continuous optimization process, some items (e.g., gender) were not gathered within all curricula.
- Step 4), quiz and evaluation. To encourage reflection on the experiment and its results, students were invited to complete an online quiz directly following data submission. For participation in the quiz, data submission was not compulsory. Conceptionally, the quiz followed the guided discovery theory and lead students through the underlying physiology of orthostasis and the BRR. The quiz consisted of 4—10 consecutive steps using different item formats (multiple choice, sorting tasks, free text, etc.) which were adapted to the respective prerequisites of each curriculum. Each question was followed by one or more brief context slides. These provided direct feedback on the correct answer(s), brief explanations and additional information. To minimize the gap between the modified and the cAST, parts of the quiz specifically focused on the changes of BP throughout the procedure. In Additional file 2, we provide exemplary insight into a selection of quiz items. Following the quiz, students were asked for open text feedback and their agreement with the statement “I perceive the AST in its current version as an advantage for physiological teaching.” on a 5-point Likert scale (1 point = no agreement; 5 points = maximal agreement).
- Step 5), aggregated results. At the end of each data collection period, experimental data of all students were plotted, analyzed and fed back to the cohort. Students were encouraged to compare their individual measurements with the means of the entire cohort and reflect on the scientifically important difference between individual and repeated measurements.

### E-learning platform & audience response system

Modular integration, communication with the students and distribution of the material was conducted using the e-learning platforms iMED-Campus (UKE, Hamburg, Germany) and moodle (Moodle Pty Ltd, West Perth, Australia). Data submission, quiz and evaluation were realized using the commercial browser based interactive platform Mentimeter (Mentimeter AB, Stockholm, Sweden). This audience response system is established at the faculty, offers a variety of question formats and allows seamless integration in digital materials.

### Evaluation and analysis

The following variables of the student reported data were used to evaluate the aims of the study.

The primary outcome was assessed by a comparison of the student reported mean rise of HR (ΔHR) during orthostasis with those in textbooks and scientific publications which were generated under professional conditions. A ΔHR of ~ 15–20 bpm has been reported under standardized conditions [40–43].

Regarding the secondary outcomes, we assessed the frequency of orthostatic symptoms, the frequency of technical issues, descriptive subgroup comparisons with respect to the primary outcome and the mean level of the students’ satisfaction (measured on a 5-point Likert-scale).

For data analysis, the data were exported from Mentimeter and further processed using Microsoft Excel (v. 2109 Microsoft, Redmond, USA). Graphs were prepared using Microsoft PowerPoint (v. 2109 Microsoft, Redmond, USA) and GraphPad Prism (v. 9.1.0, GraphPad Software, San Diego, California USA). All outcomes were analyzed solely in a descriptive manner. For the primary outcome, individual data of the entire cohort were aggregated for each of the 15 time points of the measuring protocol and plotted as time course of the cohort’s HR as mean values and 95% CIs. For calculation of the cohort’s mean HRs and 95% CIs of each experimental phase (1^st^ recumbent, standing, 2^nd^ recumbent), individual data of each phase were aggregated. The individual ∆HR was calculated from the difference of the individual mean of the standing phase and the lower individual mean of the two recumbent phases. For the subgroup comparisons of the secondary outcome, we performed the same analyses with data separated for the respective predefined subgroups.

## Results

### Cohort characteristics

The newly designed e-learning laboratory class was implemented in existing cardiovascular modules. Within one year, all enrolled students of human medicine (354, 62% female), dental medicine (64 students, 67% female), midwifery (66 students, 100% female) and pharmacy (61 students, 62% female) were strongly encouraged to participate in the voluntary class.

In the following analysis, only students who submitted a complete experimental data set were included. In total, 217 (~ 40%) of all enlisted students (n = 545) submitted complete experimental data sets (Table 1). Although curricular integration and advertisement for the laboratory class were similar in all curricula, the participation rates ranged from 20 to 73% (Table 1). Mean age of the test subjects (not necessarily the students) was 23 ± 8 years (SD; range: 15—58 years) which implicates that some students performed the experiment on another person (e.g., family members). Remarkably, nearly all students who submitted their experimental data also started the associated quiz (n = 216). Of these, 209 students completed the online quiz resulting in a very high completion rate of 96% (Table 1).

**Table 1:** Participation

**Group**	**Enlisted students**	**Complete experimental data**	**Quiz started**^a^	**Quiz ended**^a^	**Drop out**^b^
all students	545	217 (40%)	216 (40%)	209 (38%)	8 (4%)
human medicine	354	145 (41%)	145 (41%)	145 (41%)	0 (0%)
dental medicine	64	14 (22%)	14 (22%)	13 (20%)	1 (7%)
midwifery	66	13 (20%)	12 (18%)	10 (15%)	3 (23%)
pharmacy	61	45 (73%)	45 (74%)	41 (67%)	4 (9%)

^a^ only students were included who submitted complete experimental data

^b^ between submission of complete data set and last answer of the quiz

### Primary outcome

**Fig. 2:**
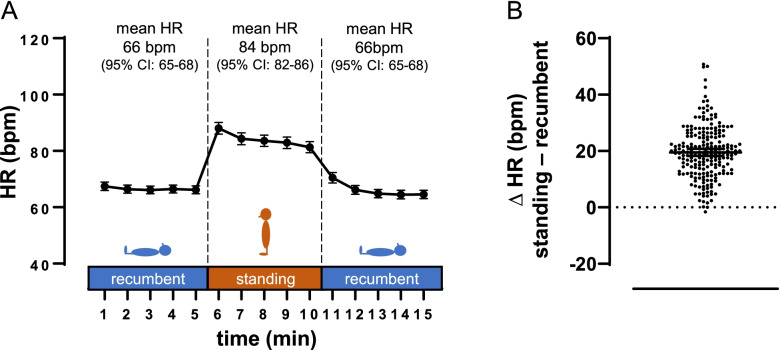
Experimental HR and ∆HR data of the whole sample. Data are given as mean and 95% CI. **A** HR of whole sample (*n* = 217) for 15 subsequent time points at the three phases of the experimental procedure. Mean and 95% CI are also given for aggregated data of each experimental phase. **B** individual ∆HR, calculated as the difference between mean standing HR and mean HR of the recumbent phase with the lower HR

The students determined an average HR of ~ 70 bpm during the 1^st^ recumbent phase (Fig. 2A). Orthostatic stress was associated with a robust increase in HR. The average ΔHR of ~ 20 bpm (~ 30%, Fig. 2A + B) is highly concordant to textbooks and previous reports by healthcare professionals using standardized methods [40–43]. On an individual level, > 90% of the students (204 of the 217 participants) reported a ΔHR of ≥ 10% while standing. Students’ data also showed a HR peak in the first minute of the standing phase (Fig. 2A). During the 2^nd^ recumbent phase, the sympathetic tonus could be expected to be reduced, e.g., due to a loss of mental tension towards the end of the experiment. Contrasting this assumption, the HRs of the two recumbent phases were similar (Fig. 2A).

### Secondary outcome

**Fig. 3:**
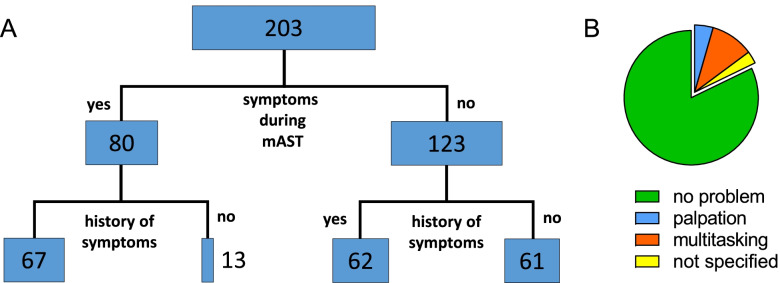
Orthostatic symptoms and technical issues. **A**), absolute numbers of students (human medicine, midwifery, pharmacy) who experienced orthostatic symptoms during the AST and those that did not. Both groups were split up further, whether a history of orthostatic symptoms was indicated or not. **B**), technical issues during the experiment reported by students (human medicine, midwifery, pharmacy, n = 202). 82% of the students did not report technical issues. 10% reported problems in handling the multitasking of e.g., measuring, documenting and relaxing, 5% of the students reported problems with the palpation of the pulse, and 3% did not further specify their problems

Students of human medicine, midwifery and pharmacy (n = 203) were asked about orthostatic symptoms during the mAST and in their medical history (Fig. 3A). Symptoms were reported by 80 students (~ 40%) during the mAST (e.g., vertigo, palpitations, visual disturbances). Of these, 67 (~ 80%) had already experienced orthostatic symptoms before (Fig. 3A). The symptoms did not correlate with the HR response (Table 2). The mAST could be performed by ~ 80% of the students of human medicine, midwifery and pharmacy without any technical or procedural issues (Fig. 3B). ~ 10% of the students felt challenged by organizing pulse measurement and documentation within the short time slot and ~ 5% by palpation of the pulse. These problems, however, did not translate into different baseline HRs or ΔHRs (Table 2).

**Table 2:** Average HR and ΔHR within the predefined subgroups

**subgroup**	**mean HR** **1**^**st**^** recumbent** **phase (bpm)**	**mean HR** **standing phase (bpm)**	**mean HR** **2**^**nd**^** recumbent** **phase (bpm)**	**∆HR**^a^ **(%)**
**symptoms during mAST**^b^ (*n* = 80)	67 (64–69)	85 (81–88)	67 (64–70)	32 (29–36)
**no symptoms during mAST**^b^ (*n* = 123)	67 (65–68)	84 (81–86)	66 (64–67)	30 (28–33)
**technical issues**^b^ (*n* = 36)	68 (65–71)	83 (78–87)	67 (63–70)	28 (24–33)
**no technical issues**^b^ (*n* = 166)	66 (65–68)	84 (82–87)	66 (64–68)	32 (30–34)
**human medicine** (*n* = 145)	66 (65–68)	85 (83–87)	66 (64–67)	33 (30–35)
**dental medicine** (*n* = 14)	65 (60–69)	79 (72–86)	65 (61–69)	25 (17–32)
**midwifery** (*n* = 13)	72 (62–82)	88 (78–99)	73 (61–86)	27 (18–36)
**pharmacy** (*n* = 45)	66 (63–69)	82 (78–86)	66 (63–69)	28 (23–32)
**females**^c^ (*n* = 47)	67 (63–71)	84 (79–89)	68 (63–72)	29 (24–33)
**males**^c^ (*n* = 11)	68 (62–73)	81 (76–87)	67 (62–72)	23 (15–31)

mean values of the students’ individual means. The data are given as mean and 95% CI

^a^ ∆HR calculated as the difference between mean standing HR and mean HR of the recumbent phase with the lower HR

^b^ data are given for students of human medicine, dental medicine, midwifery and pharmacy

^c^ data are given for students of midwifery and pharmacy

**Fig. 4:**
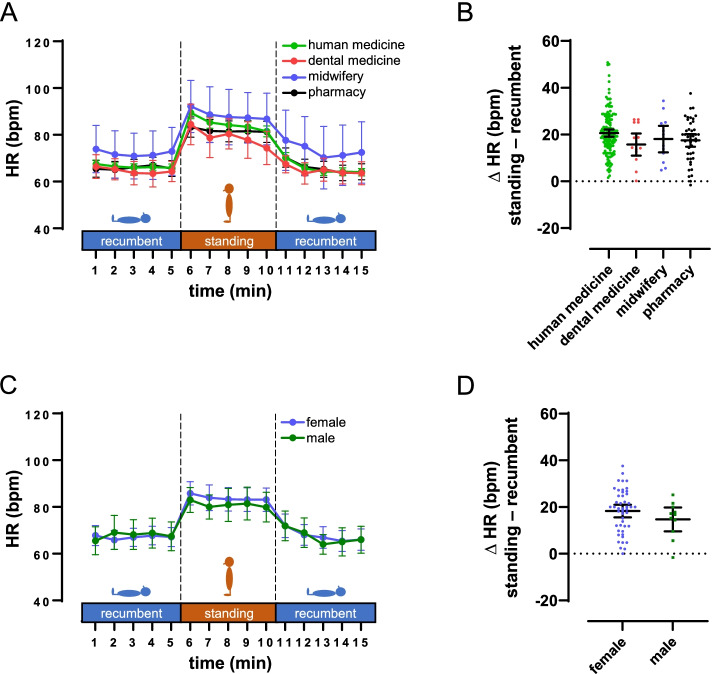
Subgroup comparisons of HR and ∆HR for careers and gender. Data are given as mean and 95% CI. A), subgroup comparison for careers (human medicine: n = 145, dental medicine: n = 14, midwifery: n = 13, pharmacy: n = 45). B), ∆HR separated for careers. C), subgroup comparison for gender (females: n = 47, males: n = 11). D), ∆HR separated for genders. ∆HR calculated as difference between mean standing HR and mean HR of the recumbent phase with the lower HR. Data are given for students of midwifery and pharmacy

One might expect that students of different study courses have different levels of interest and experience in diagnostical procedures. Therefore, it could be hypothesized that the feasibility and, therefore, the resulting HR data differ between the four involved curricula. However, in a subgroup comparison, students of all curricula documented quantitatively similar HR and ΔHR data (Fig. 4A + B, Table 2). Likewise, our data did not reveal a significant difference between male and female subjects (Fig. 4C + D, Table 2).

In the evaluation following the experimental procedure and the quiz, students reported to feel that the mAST is a gain for physiological teaching (mean approval of 4.4 (95% CI: 4.3–4.5) on a 5 points Likert-scale, n = 223). In the qualitative evaluation, students also emphasized their overall positive impression of this laboratory class. There was general enthusiasm for the practical part which enabled them to carry out a motivating experiment without a classroom event (‘I liked that it was interactive and one could do an experiment by oneself.’ and ‘The self-experiment was really fun!’). Above all, the students mentioned in their evaluations the connection between theoretical knowledge and its practical application which led to a better understanding of the underlying physiology (‘Nice combination of active and passive work assignments, especially with the self-test. Also, the explanations were very clear and understandable. My knowledge feels more consolidated’).

## Discussion

The current feasibility study aimed to investigate whether the established in-classroom laboratory class on the BRR can be transformed into a new e-learning based asynchronous laboratory class for untrained non-supervised students without medical equipment and to report the data. To this end, we derived the mAST from the cAST by omission of BP and restriction to HR measurements via radial palpation. The mAST was embedded into structuring digital materials and an online quiz. Using an audience response system, we collected the data of 217 students of four different healthcare-related curricula on epidemiology, HR, orthostatic symptoms, technical problems and students’ satisfaction.

The data was analyzed following the concept of a feasibility study. As primary outcome, we investigated the students’ correct performance of the mAST by a comparison of the reported HR responses to orthostasis with those in textbooks and scientific publications. The students reported a substantial increase of HR (ΔHR) during orthostasis. On average, the HR increased by ~ 30% from ~ 70 bpm to ~ 90 bpm. This average HR response is highly concordant with textbooks and data which were acquired in subjects using more elaborate procedures [40–43]. Over 90% of the students measured an increase in HR over 10% during orthostasis, the confidence intervals were small and, as a detail, a HR peak during the first standing measurement was distinguishable which is in line with the literature [43, 44]. The majority of the students, therefore, properly performed the orthostatic maneuver of the mAST and reliantly provoked a robust orthostatic stimulus for the BRR and correctly measured the HR with sufficiently high precision. Consequently, most students could clearly observe the didactically most relevant finding on their individual data: during standing, the BRR leads to a rise of HR. In addition, data on the secondary outcomes revealed that ~ 40% of all students and ~ 50% of the students with a positive history for orthostatic symptoms experienced orthostatic symptoms during standing. Thus, the self-performed mAST yielded excellent data on the BRR-mediated cardiovascular response and allowed a sensorial perception of the orthostatic challenge. Together, this provided a valuable experiential basis for further discussions on the underlying physiology. This includes details of the regulatory circuit of the BRR like the effects of the altered sympathetic/parasympathetic tonus, or the reasons for orthostatic symptoms. In accordance with the high-quality data, just ~ 20% of the students reported minor procedural problems during the mAST which did not affect the HR measurements. HR data also revealed no relevant differences between the four involved healthcare-related curricula. This indicates a high feasibility of this non-supervised laboratory class which argues for a transferability to other target groups.

As far as we know, the current study is the first report of HR data during an AST in a large population of young students. Therefore, it provides normal values for laboratory classes which rely on a student-performed AST. Gender effects on the baseline HR and the HR response during orthostasis are inconsistently reported in the literature [28, 44, 45]. Information on gender distribution was retrieved for 58 students (~ 25% of the cohort). In this subgroup, we could not detect a relevant gender difference.

The class followed the concept of experiential learning [32]. The didactical basis was the self-experience of the mAST which offered a variety of observations for the students, e.g., the sensory input of orthostatic symptoms or the experimental data. From this, students derived conclusions on cardiovascular physiology in the sense of a guided discovery through the quiz. Participation in the laboratory class was on a voluntary basis. In spite of this, ~ 40% of all enlisted students submitted full data and only very few students (< 5%) did not complete the subsequent quiz. This high acceptance rate and the overall positive perception among the students may be attributed to a combination of several elements, motivators and incentives included during the development of the class which in a large extent coincide with the recommendations of several authors [1, 25, 46]. The laboratory class was particularly designed for the needs of remote delivery and did not merely copy the established in-classroom laboratory [1]. Exemplarily, the BP measurement, which requires technical equipment that is unavailable to most students, was waived to reduce barriers preventing participation. On the other hand, the quiz was designed to enhance active reflection on the underlying physiology with a particular focus on the regulation of BP. By this reciprocal change in the focus of the experiment (HR) and the quiz (BP), redundancy during the laboratory class was reduced while the entire BRR was addressed. For all students, the asynchronous and non-supervised setting allowed a flexible time management [46]. To stimulate the students’ intrinsic motivation, care was taken to accentuate the high clinical and physiological relevance of the experiment during the prior communication process and within the introductory materials [46]. Moreover, the experiment addressed a central concept of cardiovascular physiology and trained a clinically relevant skill within a reasonably short course duration of ~ 60 min [1]. The self-experience with an active involvement of the students at home enhanced interactivity and helped to overcome frustration by an overload of passive digital material during the pandemic [25, 46]. The quiz constantly varied the question formats to preserve student’s vigilance. Also, correct answer(s) and short explanations were included after every item to give immediate feedback and provide additional values [46]. Care was taken to align the content, the length and the difficulty of the quiz with the curricular content and the knowledge of the students of each curriculum [46]. Finally, the inclusion of students’ own data in a data set published at the end of the collection period may allow them to relate with the cohort and increase the feeling of importance of the own experimental results.

This e-learning based class profited from the broad application of an audience response system. These systems offer a continuously increasing variety of question formats which, in a didactically guided usage, enhance fun, interactivity and diversity during teaching. Moreover, they allow acquisition of epidemiological, experimental and evaluation data of the students during the class. Typically, collecting experimental data of students is more complicated during in-classroom laboratory classes. Thus, the application of audience response systems may increase didactic and research application in the future.

### Limitations and outlook

The current study was designed to describe the conversion of the established laboratory class on the BRR response to orthostasis into a digitally embedded version that can be performed by a broad audience at home. Accordingly, we focused on the description of the new class and on a basic evaluation of the applied instrument for HR assessment, i.e., the mAST. We did not, however, test learning success. Based on the active engagement during the online quiz and the time spent on the task, we would expect positive results from participants. This should, however, be assessed in direct comparison to established in-classroom courses. The average participation rate in all curricula of 40% (range: 20–70%) may be reasonable for a voluntary class during a pandemic situation. It implicates, however, a selection bias in the reported data and illustrates a highly variable rate of participation in the different curricula. We have no insights into the reasons for non-participation. Aside from motivational aspects, students might not participate since they do not want to share their data. Or they might feel that they are not skilled enough for the task. This could be addressed by supporting video tutorials on HR measurement or by synchronous online supervision. This requires personal and technical efforts while synchronous supervision would interfere with the idea of the time-independent character of the laboratory course.

Another limitation was the omission of BP measurements which are an essential part of the cAST. This was a deliberate trade-off as the necessary technical equipment, a blood pressure cuff and a stethoscope, are not commonly available in private households. However, the changes of HR alone clearly illustrated the aim of the practical course, i.e., the regulation of the BRR. With the rapid technical evolution of smart medical devices (e.g., smartwatches), an automated measurement of HR and BP at home will become realistic for most of the students. This could also include other parameters that illustrate targets of the automatic nervous system. For instance, skin resistance could be measured to investigate sympathetic activation. A concomitant recording of the student’s ECG with a smartphone coupled device could allow a more detailed view on the regulation of cardiac electrophysiology, e.g., the QT interval. This would further enhance the educational objectives of this activity. Finally, the high quality of data and the high feasibility of the mAST strongly implicate a broad application of this class, including in non-academic settings. The course could, e.g., be implemented in lower-level education or in public education via freely accessible platforms.

## Conclusions

For this feasibility study, we transformed the established in-classroom laboratory class on the BRR into a new e-learning based asynchronous laboratory class by combining a specifically modified AST with structure providing material and a post quiz. The reported increase of HR of ~ 30% during standing was robust and in line with the data obtained from professional measurements. Students reported a high rate of orthostatic symptoms, a high technical feasibility and a high satisfaction. The HR response to orthostasis was independent of the curriculum, the gender and the occurrence of orthostatic symptoms or technical issues. Consequently, the mAST appears to be a feasible instrument for measurements of ∆HR during orthostasis for untrained and non-supervised participants. Here, we provide details on the course and previously not available reference values of HR changes during the mAST for a cohort of students. The high feasibility, the embedment into a framework allowing self-determined learning and the reduction of the cAST by omission of the BP measurement substantially reduced access barriers to this laboratory experience. This may expand the applicability of the course beyond healthcare-related curricula. With appropriate adaptations of the supporting framework, the mAST can be included to enrich teaching portfolios in virtually all levels of education. This e-learning based laboratory course, therefore, gives a broad audience access to a fundamental concept of cardiovascular physiology.

## Supplementary Information

**Additional file 1.** Instructions and experimental protocol. Stepwise instructions to the mAST and experimental protocol table as provided for the students (translated from German).

**Additional file 2.** Quiz. Table with exemplary items of the post-test quiz (translated from German).

## Data Availability

The datasets used and/or analyzed during the current study are available from the corresponding author on reasonable request.

## Abbreviations

∆HR: Rise of heart rate
AST: Active Standing Test
BP: Arterial blood pressure
BRR: Baroreceptor reflex
cAST: Conventional AST
HR: Heart rate
mAST: Modified AST

## References

[1] Choate J, Aguilar-Roca N, Beckett E, Etherington S, French M, Gaganis V (2021). International educators' attitudes, experiences, and recommendations after an abrupt transition to remote physiology laboratories. Adv Physiol Educ.

[2] Hofstein A, Lunetta VN (2004). The laboratory in science education: Foundations for the twenty-first century. Sci Educ.

[3] Pontiga F, Gaytán SP (2005). An experimental approach to the fundamental principles of hemodynamics. Adv Physiol Educ.

[4] Dohn NB, Fago A, Overgaard J, Madsen PT, Malte H (2016). Students' motivation toward laboratory work in physiology teaching. Adv Physiol Educ.

[5] Horrigan LA (2018). Tackling the threshold concepts in physiology: what is the role of the laboratory class?. Adv Physiol Educ.

[6] Burgess A, van Diggele C, Roberts C, Mellis C (2020). Tips for teaching procedural skills. BMC Med Educ.

[7] el Kharki K, Berrada K, Burgos D. Design and implementation of a virtual laboratory for physics subjects in Moroccan Universities. Sustainability. 2021;13(7):3711. 10.3390/su13073711.

[8] Ozuah PO (2002). Undergraduate medical education: thoughts on future challenges. BMC Med Educ.

[9] Walsh S, De Villiers MR, Golakai VK (2018). Introducing an E-learning solution for medical education in Liberia. Ann Glob Health.

[10] Barteit S, Jahn A, Banda SS, Bärnighausen T, Bowa A, Chileshe G (2019). E-Learning for medical education in Sub-Saharan Africa and low-resource settings: viewpoint. J Med Internet Res.

[11] Lomas L (2002). Does the development of mass education necessarily mean the end of quality?. Qual High Educ.

[12] Regmi K, Jones L (2020). A systematic review of the factors - enablers and barriers - affecting e-learning in health sciences education. BMC Med Educ.

[13] Vaona A, Banzi R, Kwag KH, Rigon G, Cereda D, Pecoraro V (2018). E-learning for health professionals. Cochrane Database Syst Rev.

[14] Ruiz JG, Mintzer MJ, Leipzig RM (2006). The impact of E-learning in medical education. Acad Med.

[15] Bhute VJ, Inguva P, Shah U, Brechtelsbauer C (2021). Transforming traditional teaching laboratories for effective remote delivery-A review. Educ Chem Eng.

[16] Sinclair PM, Levett-Jones T, Morris A, Carter B, Bennett PN, Kable A (2017). High engagement, high quality: A guiding framework for developing empirically informed asynchronous e-learning programs for health professional educators. Nurs Health Sci.

[17] Gamage KAA, Wijesuriya DI, Ekanayake SY, Rennie AEW, Lambert CG, Gunawardhana N (2020). Online delivery of teaching and laboratory practices: continuity of university programmes during COVID-19 pandemic. Educ Sci.

[18] Bishop ZK, Howard T, Lazari P, Taylor B, Trend P, Funnell A (2021). Student experiences of practical activities during the COVID-19 Pandemic IEEE Global Engineering Education Conference (IEEE EDUCON); 2021 Apr 21–23.

[19] Goodacre CJ, Younan R, Kearbey V, Fitzpatrick M (2021). An educational experiment resulting from COVID-19: the use of at-home waxing and webinars for teaching a 3-week intensive course in tooth morphology to first year dental students. J Prosthodont.

[20] Costabile M (2021). Design, implementation, and assessment of an interactive simulation to teach undergraduate immunology students hemolytic disease of the newborn. Adv Physiol Educ.

[21] Pasricha ND, Haq Z, Ahmad TR, Chan L, Redd TK, Seitzman GD (2020). Remote corneal suturing wet lab: microsurgical education during the COVID-19 pandemic. J Cataract Refract Surg.

[22] Colthorpe K, Ainscough L (2021). Do-it-yourself physiology labs: Can hands-on laboratory classes be effectively replicated online?. Adv Physiol Educ.

[23] Longhurst GJ, Stone DM, Dulohery K, Scully D, Campbell T, Smith CF (2020). Strength, Weakness, Opportunity, Threat (SWOT) analysis of the adaptations to anatomical education in the United Kingdom and Republic of Ireland in response to the Covid-19 pandemic. Anat Sci Educ.

[24] Amer MG, Nemenqani DM (2020). Successful use of virtual microscopy in the assessment of practical histology during pandemic COVID-19: a descriptive study. J Microsc Ultrastruct.

[25] Lellis-Santos C, Abdulkader F (2020). Smartphone-assisted experimentation as a didactic strategy to maintain practical lessons in remote education: alternatives for physiology education during the COVID-19 pandemic. Adv Physiol Educ.

[26] Smit AA, Halliwill JR, Low PA, Wieling W (1999). Pathophysiological basis of orthostatic hypotension in autonomic failure. J Physiol.

[27] Aydin AE, Soysal P, Isik AT (2017). Which is preferable for orthostatic hypotension diagnosis in older adults: active standing test or head-up tilt table test?. Clin Interv Aging.

[28] Okamura S, Sairaku A, Tokuyama T, Okubo Y, Ikeuchi Y, Miyauchi S (2021). Peripheral arterial tone during active standing. Pflugers Arch.

[29] Blanc JJ (2013). Clinical laboratory testing: what is the role of tilt-table testing, active standing test, carotid massage, electrophysiological testing and ATP test in the syncope evaluation?. Prog Cardiovasc Dis.

[30] Fanciulli A, Campese N, Wenning GK (2019). The Schellong test: detecting orthostatic blood pressure and heart rate changes in German-speaking countries. Clin Auton Res.

[31] Finucane C, van Wijnen VK, Fan CW, Soraghan C, Byrne L, Westerhof BE (2019). A practical guide to active stand testing and analysis using continuous beat-to-beat non-invasive blood pressure monitoring. Clin Auton Res.

[32] Kolb D, Boyatzis R, Mainemelis C, Sternberg RJ, Zhang LF (2000). Experiential learning theory: previous research and new directions, in perspectives on thinking, learning and cognitive styles. Perspectives on cognitive, learning, and thinking styles.

[33] Lahrmann H, Cortelli P, Hilz M, Mathias CJ, Struhal W, Tassinari M (2006). EFNS guidelines on the diagnosis and management of orthostatic hypotension. Eur J Neurol.

[34] Matsushima R, Tanaka H, Tamai H (2004). Comparison of the active standing test and head-up tilt test for diagnosis of syncope in childhood and adolescence. Clin Auton Res.

[35] Hartwig MS, Cardoso SS, Hathaway DK, Gaber AO (1994). Reliability and validity of cardiovascular and vasomotor autonomic function tests. Diabetes Care.

[36] Thijs RD, Brignole M, Falup-Pecurariu C, Fanciulli A, Freeman R, Guaraldi P (2021). Recommendations for tilt table testing and other provocative cardiovascular autonomic tests in conditions that may cause transient loss of consciousness : Consensus statement of the European Federation of Autonomic Societies (EFAS) endorsed by the American Autonomic Society (AAS) and the European Academy of Neurology (EAN). Clin Auton Res.

[37] Voelliger CM, VanderZwan KJ, Coyne EP, Hu Y, Shammas NW, Lisius K (2021). Education of Self-Radial Pulse Palpation and Atrial Fibrillation Signs and Symptoms. J Community Health Nurs.

[38] Hollerbach AD, Sneed NV (1990). Accuracy of radial pulse assessment by length of counting interval. Heart Lung.

[39] Kobayashi H (2013). Effect of measurement duration on accuracy of pulse-counting. Ergonomics.

[40] Levick JR (2010). An introduction to cardiovascular physiology.

[41] Smith JJ, Bush JE, Wiedmeier VT, Tristani FE (1970). Application of impedance cardiography to study of postural stress. J Appl Physiol.

[42] Jacobsen TN, Morgan BJ, Scherrer U, Vissing SF, Lange RA, Johnson N (1993). Relative contributions of cardiopulmonary and sinoaortic baroreflexes in causing sympathetic activation in the human skeletal muscle circulation during orthostatic stress. Circ Res.

[43] van Wijnen VK, Finucane C, Harms MPM, Nolan H, Freeman RL, Westerhof BE (2017). Noninvasive beat-to-beat finger arterial pressure monitoring during orthostasis: a comprehensive review of normal and abnormal responses at different ages. J Intern Med.

[44] Finucane C, O'Connell MD, Fan CW, Savva GM, Soraghan CJ, Nolan H (2014). Age-related normative changes in phasic orthostatic blood pressure in a large population study: findings from The Irish Longitudinal Study on Ageing (TILDA). Circulation.

[45] Hnatkova K, Šišáková M, Smetana P, Toman O, Huster KM, Novotný T (2019). Sex differences in heart rate responses to postural provocations. Int J Cardiol.

[46] de Leeuw RA, Westerman M, Scheele F (2017). Quality indicators for learner-centered postgraduate medical e-learning. Int J Med Educ.

