# Supplementary material for: The baroreceptor reflex brought to life outside the classroom – an e-learning based asynchronous laboratory class using a non-supervised modified Active Standing Test
Source: BMC Med Educ. 2022 Jul 1;22:515. doi: 10.1186/s12909-022-03573-7 (PMC9250187; doi:10.1186/s12909-022-03573-7)
Supplement: Supplementary file 1 — Additional file 1. Instructions and experimental protocol. Stepwise instructions to the mAST and experimental protocol table as provided for the students (translated from German). [file 12909_2022_3573_MOESM1_ESM.pdf]

Instructions for the modified AST

Duration:

approximately 20 minutes

Preparation:

- stopwatch / timer
- experimental protocol table (printed or digitally editable)
- pen (if needed)
- quiet room with bed or sofa (increased sympathetic activity distorts the measurement)
- Please do not talk during the experiment!
- optional: 2nd person on whom you can perform the experiment, or who can perform the experiment on you. Performing the experiment alone may be more challenging, but it works!
- practice measurement of your heart rate (HR) before the experiment:
  - locate your radial pulse
  - count pulse beats on radial artery for 15 s
  - multiply by the factor 4

Experimental procedure (step-by-step):

1) **Relaxation (5 min)**
   - lie down for 5 min
   - do not take measurements
   - the goal is a high vagal tone → so be relaxed

2) **1st recumbent phase (5 min)**
   - measure your heart rate every full minute for 5 times
   - document your heart rate in the table on the next page

3) **Standing phase (5 min)**
   - stand up swiftly and avoid unnecessary movement (muscle pump)
   - stand calmly, without "wobbling back and forth" on the legs (muscle pump)
   - immediately start the 1st measurement
   - repeat measurement every full minute until you have 5 measurements
   - document your heart rate in the table on the next page

4) **2nd recumbent phase (5 min)**
   - lie down
   - immediately start the 1st measurement
   - repeat measurement every full minute until you have 5 measurements
   - document your heart rate in the table on the next page

Experimental protocol

1st recumbent phase:

measurement	1	2	3	4	5
heart rate (bpm)					

Standing phase:

measurement	1	2	3	4	5
heart rate (bpm)					

2nd recumbent phase:

measurement	1	2	3	4	5
heart rate (bpm)					
