# Supplementary material for: The baroreceptor reflex brought to life outside the classroom – an e-learning based asynchronous laboratory class using a non-supervised modified Active Standing Test
Source: BMC Med Educ. 2022 Jul 1;22:515. doi: 10.1186/s12909-022-03573-7 (PMC9250187; doi:10.1186/s12909-022-03573-7)
Supplement: Supplementary file 2 — Additional file 2. Quiz. Table with exemplary items of the post-test quiz (translated from German). [file 12909_2022_3573_MOESM2_ESM.pdf]

Additional file 2: quiz

intention	question			subsequent or independent context slide	
	format	text	answers to choose	text	image
guided discovery (through experimental results)	multiple choice (1 option to choose)	Which changes occurred to your heart rate in the standing phase (compared to recumbent 1st phase)?	A) drop of 10 /min or more<br>B) no big change (less than 10/min)<br>C) rise of 10 /min or more	correct: C)<br>normal values<br>transfer: role of the increase in HR for blood pressure	example of normal heart rate course during procedure in a healthy person
reflection on omitted parameter (blood pressure)	open ended	In the "complete" AST, besides heart rate, blood pressure is assessed. Which changes would you expect to systolic and diastolic blood pressure during the standing phase?		correct: slight rise<br>normal values<br>side note on involved cardiovascular changes	mean course of systolic and diastolic blood pressure during the procedure in healthy persons
additional clinical information (pathological courses of HR and blood pressure)	context slide			pathomechanisms of orthostatic hypotension<br>symptoms<br>thresholds	course of HR, systolic and diastolic blood pressure of A) healthy person B) patient with orthostatic hypotension
transfer (cause of experienced symptoms and/or cardiovascular response)	scales with sliders	What happens to hydrostatic pressure in different body sites during standing phase? (in relation to hydrostatic indifference point)	slider options: drops/unchanged/rises<br>items (one slider each):<br>A) hydrostatic pressure above HIP*<br>B) intravascular volume above HIP*<br>C) hydrostatic pressure on HIP*<br>D) hydrostatic pressure below HIP*<br>E) intravascular volume below HIP*	correct: A) drops, B) drops, C) unchanged, D) raises, E) raises<br>brief review of concerning slides from lecture	textbook image on changes of hydrostatic pressure during standing position

transfer from HR to other physiological mechanisms which were not addressed in experiment (e.g. total peripheral resistance)	multiple choice (1 option to choose)	To prevent a drop of blood pressure, heart rate is being raised. Which other circulatory parameters become significantly increased, too?	A) total peripheral resistance<br>B) vagal activity<br>C) outward filtration in capillary beds of organs above HIP*<br>D) respiratory rate<br>E) oxygen saturation of the blood	correct: A)<br>effects of total peripheral resistance on blood pressure	mean course of total peripheral resistance during procedure in healthy persons
integration / context of learned physiological processes	sorting task	Now, let us bring everything together! Please sort the following steps into the correct chronology as they occur under orthostatic stress.	A) quick raise from the recumbent position<br>B) rise of contractility, heart rate and total peripheral resistance<br>C) stabilization of cranial perfusion<br>D) increase or at least stabilization of mean arterial pressure<br>E) disinhibition of sympathetic activity<br>F) hydrostatic pressure on heart level drops<br>G) decreased stimulation of baroreceptors in carotid sinus and aortic bodies<br>H) drop of diastolic ventricular filling and end-diastolic volume<br>I) Frank-Starling mechanism: drop of stroke volume		correct order: A), F), H), I), G), E), B), D), C)
view on further cardiovascular regulation processes (sports)	multiple choice (more options to choose)	You decide to end this day actively by doing a bicycle tour. To forget the confusing content of this seminar, you peddle quite hard.<br>Which statements on the cardiovascular response are correct?	A) the baroreceptors prevent your blood pressure from changing<br>B) perfusion of your skeletal muscles increases<br>C) your skin perfusion increases<br>D) your stroke volume rises		correct: B), C), D)<br>brief explanation on reason for each answer
shift to a pathological cardiovascular reaction (hemorrhage)	multiple choice (more options to choose)	Unfortunately, you thought to much about the physiology you learned today and suffer an accident. You lose 500 ml of blood. Which effects or counter regulations occur?	A) drop of venous return<br>B) rise of heart rate<br>C) increased stimulation of arterial baroreceptors<br>D) decrease of vasoconstriction in the body circulation<br>E) reduction of cardiac contractility (negative inotropic effect)<br>F) decrease of stroke volume		correct: A), B)<br>brief explanation on reason for each answer

* hydrostatic indifference point
